# Exploring factors influencing health-related quality of life in adult females with injuries: a cross-sectional nationwide study

**DOI:** 10.3389/fpubh.2023.1248583

**Published:** 2023-10-04

**Authors:** YeunHee Kwak, Insil Jang

**Affiliations:** Department of Nursing, Chung-Ang University, Seoul, Republic of Korea

**Keywords:** women, injury, quality of life, middle age, health promotion

## Abstract

**Objectives:**

This study aimed to identify the types of injury experiences affecting adult females and the associations between injury experiences and health-related quality of life.

**Methods:**

This study used a secondary analysis of cross-sectional data from the Korea National Health and Nutrition Examination Survey, which employed a stratified multistage probability sampling design to obtain nationally representative data. Health-related quality of life was measured with the EuroQol Five-Dimension and consisted of mobility, self-care, usual activity, pain/discomfort, and anxiety/depression.

**Results:**

Among women aged 19–64, the prevalence of injury experience was 5.9%. Women’s injury experience varied by age, subjective health perception, menstruation details, osteoporosis status, and osteoarthritis status. The following injury experience–related factors were significantly associated with health-related quality of life: self-care (odds ratio [OR] = 0.32, 95% confidence interval [CI]: 0.17–0.60), usual activity (OR = 0.43, 95% CI: 0.27–0.70), and pain/discomfort (OR = 0.56, 95% CI: 0.44–0.72).

**Conclusion:**

Health-related quality of life among adult women was significantly associated with injury experience. The results of this study highlight risk factors for injury that are associated with aging, such as menopause, osteoporosis, and osteoarthritis. Accurate assessments of pain and discomfort, as well as daily activities, are essential for designing effective interventions for injured women. Tailored training and education can promote self-management and improve outcomes for recovery.

## 1. Introduction

Annually, more than 4.4 million individuals (approximately 8% of the population) worldwide succumb to injuries, with injuries accounting for about 8.7% of all deaths in South Korea in 2020 (1, 2). Injuries often require emergency services, treatment, and rehabilitation, and they can result in permanent disability or even death. Injury refers to bodily damage caused by external factors other than disease. In other words, injuries are the harmful physical sequelae of intentional or unintentional accidents, including trauma and poisoning (3). An injury is defined as the adverse outcomes on both physical and mental health that arise due to deliberate or unintentional accidents (1). Injuries are more likely to occur in the underprivileged as a result of the inequitable structure of healthcare services, and the lower the socioeconomic level, the higher the risk of injury (4, 5). It is often difficult to recover completely without permanent impairments or complications after an injury, and injuries are accompanied by high medical costs and deterioration of quality of life in physical and psychological terms. Moreover, without appropriate medical or surgical care after injury, the incidence and severity of complications increase, and lifelong physical and mental hardship can be the result (6).

In the case of road traffic injuries, age, gender, socioeconomic status, injury severity, injury type, and post-traumatic stress disorder have been shown to be influencing factors that decrease quality of life (7, 8). Even the experience of a fall with no significant sequelae leads to a lack of self-confidence in performing daily life, which causes side effects, such as activity self-limitation, social isolation, stress, anxiety, and depression, leading to a decrease in overall quality of life (9, 10). Additionally, various interventions for fall prevention have been shown to be positive mediators of the relationship between injury occurrence and quality of life (11–13). Notably, injuries have controllable risk factors, and they can sometimes be prevented by applying tailored interventions to high-risk individuals. In particular, injuries may affect men and women differently due to differences in anatomy, physiology, behavior, and societal norms. Gender differences exist in the incidence, severity, and consequences of injuries (14–16). It is important to evaluate injury risk factors in detail as female sex and older age may be associated with lower health-related quality of life (HRQOL) after injury (17). Understanding gender differences can inform injury prevention strategies and improve outcomes for both men and women.

HRQOL refers to health-related elements that encompass not only physical health status but also subjective concepts, such as social functioning, roles, and emotional states (18). In other words, it is a more comprehensive concept than an individual’s health status and well-being, and it is emerging as an important concept that connects health and quality of life. Therefore, the measurement of HRQOL is being used as an important index to evaluate the health and well-being of not only patients but also the general public. Through the measurement of HRQOL, health service needs are identified and used as important data for health promotion programs, national health policy establishment, and performance monitoring. Equity of health and medical services and improvement of quality of life are major directions of national health policy (19).

Using national survey data, this study aimed to identify controllable risk factors for injury and provide basic data for developing an injury prevention strategy to improve quality of life and maintain equity. Based on data from the Korea National Health and Nutrition Examination Survey (KNHANES) from 2016 through 2020, targeting women between the ages of 19 and 64 years, the specific objectives of this study were as follows: (1) identify injury experiences and types of injury, (2) identify variation in the occurrence of injuries according to the characteristics of the participants, (3) identify the HRQOL according to injury experience, and (4) identify the relationship between the injury experience and the HRQOL.

## 2. Methods

### 2.1. Study design

This cross-sectional study was a secondary analysis of the 2016–2020 KNHANES data, investigating the relationship between injury experience and HRQOL among Korean women aged 19 to 64 years. The KNHANES is a nationally representative, cross-sectional survey targeting non-institutionalized Korean people.

### 2.2. Study setting and participants

The KNHANES has been undertaken by the Korea Disease Control and Prevention Agency (KDCA) to summarize Korean health and nutrition data since 1998. The 2016–2020 KNHANES data were reviewed and approved by the KDCA’s institutional review board (approval no. 2013-07CON-03-4C, 2013-12EXP-03-5C). Informed consent was obtained from all participants when the 2016–2020 KNHANES was conducted. The KNHANES consists of a health survey, nutrition survey, and physical examination survey. The health survey and nutrition survey were conducted through one-on-one interviews and self-reporting methods, and the medical examination survey was conducted by a specialized survey team at the KDCA. A sample was extracted to represent the Korean people using a stratified, multistage, clustered, probability design targeting the entire population. This study analyzed the data of women aged 19 to 64 (*N* = 6,540) from among the KNHANES (2016–2020) data (*n* = 24,269). Item-specific missing values were excluded from the analysis. Three hundred fifty-two women (5.9%) had at least one injury experience, and 5,603 (94.1%) had no injury experiences.

### 2.3. Study variables

#### 2.3.1. Sociodemographic and health-related characteristics

The following sociodemographic and health-related characteristics were recorded: age, education status, household size (living alone or not), occupation, stress level, subjective health perception, smoking status, and alcohol consumption, and sleep time. The household economic level was classified based on the equivalent income (average monthly household income/ $\sqrt{\mathrm{number of family members}}$ ). Values in the lowest 25% of the data were designated as the first quartile, and those in the subsequent three levels (25% each) were designated as the second, third, and fourth quartiles, respectively. Household size was analyzed as a dichotomous variable (living alone or not), as were occupation (currently employed or not), smoking status (current smoker or not), alcohol consumption (more than once a month in the past year or not). Stress levels were evaluated according to responses to the question, “How much stress do you usually feel in your daily life?” Responses of “feel so much” and “feel a lot” were classified as “high,” and “feel a little” and “feel very little” were categorized as “low.” Among the answers to the question about subjective health status, “How do you usually think about your health?,” “very good” and “good” were classified as “good,” “average” was classified as “average,” and “poor” and “very poor” were classified as “bad.” Sleep time was classified based on a cut-off of 7 h of sleep per day (<7 h or ≥ 7 h).

The following health-related characteristics were recorded: menstruation details, waist circumference (WC), body mass index (BMI), osteoporosis status, arthritis status (including osteoarthritis and rheumatoid arthritis), and depressive symptom. Height, WC, and weight were documented to the nearest 0.1 cm, 0.1 cm, and 0.1 kg, respectively. End-tidal WC was measured at the narrowest point between the lower border of the rib cage and the iliac crest using a handheld stadiometer ruler (Se-ca 225, Seca, Germany). The weight was measured using a calibrated balance beam scale (GL-6000-20; G-tech, Seoul, Korea). BMI was calculated based on the measurements, and anthropometric measurements were similarly performed by well-trained examiners throughout the study period. The osteoporosis, osteoarthritis, and rheumatoid arthritis variables were dichotomous (those diagnosed by a doctor vs. not) (20). Depressive symptom was classified as “yes” or “no” based on responses to the question, “Have you ever felt sad or hopeless enough to interfere with your daily life for more than 2 weeks in a row in the past year?”

#### 2.3.2. Injury experience

Injury experience was classified as “yes” and “no” according to responses to the question, “Have you ever had an accident or poisoning that required treatment at a hospital or clinic or emergency room (including public health center) in the past year?” This did not include injuries that were treated by self-medication or over-the-counter medications. Nine options were selectable to indicate the cause of injury: (traffic accident, falls/slip, impingement, laceration or stab wound/amputation/penetrating wound, burns, suffocation, drowning, poisoning, and others). Additionally, the intentionality of injury was divided into three categories: accident, intentional self-harm, or violence by others. Overall, injury was defined as intentional or unintentional harm inflicted on the body or mind (1).

#### 2.3.3. Health-related quality of life

For HRQOL, the EQ-5D (EuroQol Five-Dimension) and EQ-5D index, developed by the EuroQol, group were used (21). The EQ-5D is a tool used to measure HRQOL, which was developed to measure overall health in a simple way for the purpose of clinical and economic evaluation. The EQ-5D is a widely used survey instrument for describing HRQOL status (22). The EQ-5D consists of five dimensions: mobility, self-care, usual activity, pain/discomfort, and anxiety/depression. For each dimension, respondents can select one of three options: “no problems,” “some problems,” or “severe problems.” In this study, “some problems” and “severe problems” were grouped together as “yes.” In this study, the EQ-5D index value was weighted by KDCA, which expresses health status as a value from −0.171 to 1, taking into account the characteristics of Koreans. The EQ-5D’s score ranges from −1 to 1, with scores closer to 1 reflecting better quality of life. The weights were as follows: EQ-5D = 1 − (0.05 + 0.096 × M2 + 0.418 × M3 + 0.046 × SC2 + 0.136 × SC3 + 0.051 × UA2 + 0.208 × UA3 + 0.037 × PD2 + 0.151 × PD3 + 0.043 × AD2 + 0.158A × D3 + 0.05 × N3) (23).

### 2.4. Data analysis

SAS, version 9.3 (SAS Institute, Cary, NC, United States) was used to run a complex sample design to analyze the survey data, ensuring appropriate sampling weights and nationally representative estimates. For the sociodemographic and health-related characteristics, means and standard deviations or frequencies were calculated when analyzing data, without using weights. Variations in injury experience in terms of demographic and health-related characteristics, as well as HRQOL among participants, were analyzed using *t*-tests and the *χ*^2^ test. To confirm the associations of injury experience with EQ-5D index values and EQ-5D outcomes, respectively, univariate and multivariate logistic regression analyses were conducted after controlling for the subjects’ demographic characteristics, with significant covariates from the univariate analysis entered into multivariate analysis. Statistical significance was indicated by *p* values <0.05.

## 3. Results

### 3.1. Injury experience of participants

The participants were divided into young women (19–44 years old) and middle-aged women (45–64 years old) (Table 1). The overall injury rate was 5.9%, and the middle-aged women had significantly more injury experience than the younger women (*p* = 0.004). Among the participants who experienced falls/slips as causes of injury, 31.2% were younger women, and 68.8% were middle-aged women (*p* < 0.001). Most were accidents, and middle-aged women had significantly more instances of bed rest or missed work resulting from injuries.

**Table 1:** Types of injury experiences among Korean women (*N* = 6,540).

Variable		19–44 years (*n* = 3,376)	45–64 years (*n* = 3,164)	Total (*n* = 6,540)	***χ ***^**2**^ (*p*)
		*n* (%)			
Injury experiences	Yes	157 (5.1)	198 (6.8)	352 (5.9)	8.09 (0.004)
	No	2,889 (94.1)	2,714 (93.2)	5,603 (94.1)	
Cause of injury	Traffic accident	52 (50.5)	51 (49.5)	104 (100.0)	0.02 (0.892)
	Fall/slip	34 (31.2)	75 (68.8)	109 (100.0)	18.89 (<0.001)
	Impingement	26 (47.3)	29 (52.7)	55 (100.0)	0.41 (0.517)
	Laceration/stab wound, amputation, penetrating wound	14 (56.0)	11 (44.0)	25 (100.0)	0.19 (0.660)
	Others	36 (46.8)	41 (53.2)	77 (100.0)	0.15 (0.701)
Intentionality	Accident	154 (43.9)	197 (56.1)	351 (100.0)	8.91 (0.003)
	Intentional self-harm	0	1	1	1.07 (0.301)
	Violence by others	1	0	1	0.94 (0.333)
Bed-ridden	Yes	59 (1.8)	80 (2.5)	139 (2.1)	4.77 (0.028)
	No	3,317 (98.2)	3,084 (97.5)	6,401 (97.9)	
Missed work	Yes	97 (2.9)	124 (3.9)	221 (3.4)	5.47 (0.014)
	No	3,279 (97.1)	3,040 (96.1)	6,319 (96.6)	

### 3.2. Differences in sociodemographic characteristics, health-related characteristics, and HRQOL according to injury experience

Differences in sociodemographic and health-related characteristics according to the presence or absence of injury are shown in Tables 2, 3. Participants with injury experience were significantly older (*p* = 0.025) and had significantly better self-perception of their health status (*p* = 0.021) than participants without injury experience (Table 2). Participants with injury experience also had significantly higher rates of menopause (*p* = 0.005), osteoporosis (*p* < 0.001), and osteoarthritis (*p* = 0.006) (Table 3).

**Table 2:** Differences in sociodemographic characteristics according to injury experiences among Korean women (*N* = 6,540).

Variable		Injury experience, mean ± SD or n (%)		***χ ***^**2**^ or *t* (*p*)
		Yes (*n* = 352)	No (*n* = 6,188)	
Age (years)		45.0 ± 13.2	43.4 ± 12.7	−2.25 (0.025)
Education	≤Elementary school	30 (8.6)	418 (7.5)	3.04 (0.386)
	Middle school	37 (10.5)	463 (8.3)	
	High school	126 (35.9)	2054 (36.7)	
	≥College	158 (45.0)	2,661 (47.5)	
Household income	Lower	44 (12.5)	469 (8.4)	7.47 (0.053)
	Lower middle	80 (22.8)	1,295 (23.2)	
	Middle high	101 (28.8)	1759 (31.4)	
	High	126 (35.9)	2069 (37.0)	
Living alone	Yes	33 (9.4)	405 (7.2)	2.24 (0.134)
	No	319 (90.6)	5,198 (92.8)	
Occupation	Yes	208 (59.3)	3,454 (61.7)	0.83 (0.361)
	No	143 (40.7)	2,144 (38.3)	
Stress level	High	113 (32.3)	1,642 (29.4)	1.36 (0.243)
	Low	237 (67.7)	3,952 (70.6)	
Subjective health perception	Good	91 (25.9)	1758 (31.4)	7.76 (0.021)
	Average	195 (55.4)	3,042 (54.3)	
	Bad	66 (18.7)	803 (14.3)	
Current smoker	Yes	29 (8.2)	325 (5.8)	3.52 (0.060)
	No	323 (91.8)	5,278 (94.2)	
Current alcohol consumption	Yes	190 (54.0)	3,303 (58.9)	3.38 (0.066)
	No	162 (46.0)	2,300 (41.1)	
Sleep time	<7 h	205 (58.2)	3,505 (62.6)	2.63 (0.105)
	≥7 h	147 (41.8)	2098 (37.4)	

SD, standard deviation.

**Table 3:** Differences in health-related characteristics according to injury experiences among Korean women (*N* = 6,540).

Variable		Injury experience, mean ± SD or n (%)		***χ ***^**2**^ or *t* (*p*)
		Yes (*n* = 352)	No (*n* = 6,188)	
Menstruation	Yes	206 (59.2)	3,683 (66.6)	8.02 (0.005)
	No	142 (40.8)	1847 (33.4)	
Waist circumference(cm)		74.2 ± 6.3	73.8 ± 6.2	−1.24 (0.215)
BMI	BMI < 18.5	20 (5.7)	403 (7.2)	4.98 (0.082)
	18.5 < BMI < 24.9	283 (80.4)	4,615 (82.4)	
	BMI > 25	49 (13.9)	585 (10.4)	
Osteoporosis	Yes	36 (10.2)	286 (5.1)	16.99 (<0.001)
	No	316 (89.8)	5,317 (94.9)	
Osteoarthritis	Yes	38 (10.8)	387 (6.9)	7.56 (0.006)
	No	314 (89.2)	5,216 (93.1)	
Rheumatoid arthritis	Yes	4 (1.1)	98 (1.7)	0.74 (0.390)
	No	348 (98.9)	5,505 (98.3)	
Depressive symptom	Yes	19 (16.4)	220 (11.9)	2.04 (0.153)
	No	97 (83.6)	1,628 (88.1)	

BMI, body mass index; SD, standard deviation.

Participants with injury experience had a significantly lower mean EQ-5D index (*p* < 0.001), more difficulty in self-care (*p* < 0.001), more difficulty in usual activities (*p* < 0.001), and more pain/discomfort (*p* < 0.001) (Table 4).

**Table 4:** Differences in HRQOL according to injury experiences among Korean women (*N* = 6,540).

Variable		Injury experience, mean ± SD or n (%)		***χ ***^**2**^ or *t* (*p*)
		Yes (*n* = 352)	No (*n* = 6,188)	
Mobility	No	26 (7.4)	288 (5.1)	3.35 (0.067)
	Yes	326 (92.6)	5,315 (94.9)	
Self-care	No	13 (3.7)	61 (1.1)	18.30 (<0.001)
	Yes	339 (96.3)	5,542 (98.9)	
Usual activity	No	23 (6.5)	147 (2.6)	18.26 (<0.001)
	Yes	329 (93.5)	5,456 (97.4)	
Pain/discomfort	No	110 (31.3)	1,081 (19.3)	29.59 (<0.001)
	Yes	242 (68.7)	4,522 (80.7)	
Anxiety/depression	No	41 (11.6)	494 (8.8)	3.25 (0.072)
	Yes	311 (88.4)	5,109 (91.2)	
EQ-5D index		0.95 ± 0.08	0.97 ± 0.07	4.22 (<0.001)

EQ-5D, the EuroQol Five-Dimension; SD, standard deviation.

### 3.3. Relevance of HRQOL according to injury experience

Table 5 presents data reflecting the association between HRQOL and the presence or absence of injury experience. The analysis adjusted for age, education, living alone, household income, BMI, occupation, physical activity, smoking status, and alcohol consumption. Wald of overall model fit is 55.06 (*p* < 0.001). Even after adjustments for these covariates, self-care (OR = 0.32, *p* < 0.001), the ability to perform usual activities (OR = 0.43, *p* < 0.001), and pain/discomfort (OR = 0.56, *p* < 0.001) were significantly associated with HRQOL. In other words, women who had experienced an injury were negatively affected in the order of self-care, usual activity, and pain/discomfort among HRQOL compared to women who had not experienced an injury.

**Table 5:** Associations between HRQOL and injury experiences among Korean women (*N* = 6,540).

Variable	ß	SE	Injury experiences, OR (95%CI)		Wald	*p*
			Yes (*n* = 352)	No (*n* = 6,188)		
*Intercept*	2.71	0.58			21.68	<0.001
*EQ-5D index*						
Mobility (yes)	−0.12	0.11	0.79 (0.51–1.22)	1	1.15	0.284
Self-care (yes)	−0.57	0.16	0.32 (0.17–0.60)	1	12.38	<0.001
Usual activity (yes)	−0.42	0.12	0.43 (0.27–0.70)	1	11.71	<0.001
Pain/discomfort (yes)	−0.29	0.06	0.56 (0.44–0.72)	1	22.04	<0.001
Anxiety/depression (yes)	−0.12	0.09	0.79 (0.56–1.12)	1	1.71	0.191
Age	−0.01	0.01	0.99 (0.98–1.00)	1	1.43	0.232
Education (high school)	0.01	0.10	1.04 (0.81–1.34)	1	0.01	0.909
Living alone (yes)	−0.05	0.11	0.89 (0.61–1.33)	1	0.29	0.588
Household income (lower middle)	0.11	0.09	1.06 (0.81–1.34)	1	1.18	0.276
Body mass index	−0.02	0.02	0.98 (0.94–1.02)	1	0.67	0.412
Occupation (yes)	0.02	0.06	1.04 (0.83–1.31)	1	0.14	0.706
Physical activity (yes)	0.01	0.02	1.15 (0.92–1.43)	1	1.57	0.209
Smoking status (yes)	0.21	0.11	1.52 (1.01–2.30)	1	3.97	0.046
Alcohol consumption (yes)	−0.09	0.06	0.84 (0.68–1.05)	1	2.29	0.130
Wald (*p*) 55.06 (<0.001)						

OR, odds ratio; CI, confidence interval; SE, standard error.

* Adjusted for age, education, living alone, household income, body mass index, occupation, physical activity, smoking status, and alcohol consumption.

## 4. Discussion

This study investigated the degree of injury experience among Korean women, as well as the relationship between injury experience and HRQOL. Most injuries were unexpected accidents, and the patterns of injury mechanisms varied according to age: as age increased, the number of falls increased. Additionally, as age increased, there were more cases wherein activities were stopped because of injury. Injuries, such as injuries incurred from road traffic crashes, occur frequently in persons who are older, of the female gender, and of lower socioeconomic status (7). In particular, in this study, injury experience was significantly higher among women who were not menstruating, that is, middle-aged women who had reached menopause, suggesting that injury experience increases with age. Previous studies have indicated that injury is more likely to occur in vulnerable groups, such as older people as well as those with low educational levels and low income (4, 24). Regarding low educational level and low income, if timely medical services are not available, the probabilities of complications and severe infirmity increase (4, 6). Therefore, educational and intervention programs should be developed for injury prevention for middle-aged women.

People with higher subjective perception of their health status generally have higher physical activity levels (25). Additionally, the incidence of falls has been shown to be higher among those with low subjective health perception (24). People who are highly aware of their health status tend to actively implement self-management measures, such as regular physical activity, to maintain or improve their health. Positive thoughts, along with accurate recognition of health status, have been associated with self-efficacy, self-care, and motivation (26, 27). Therefore, facilitating positive self-perception regarding health status can help prevent functional weakness.

Worsening of osteoporosis is associated with a decrease in physical activity. Osteoarthritis starts with mild pain, but deterioration due to wear and tear restricts activity due to aggravation of pain (13, 28). The rates of osteoporosis and osteoarthritis increase with age and are higher among women. These conditions adversely impact HRQOL, as they limit independent daily activities. Both conditions are also linked to an increased risk of falls and fractures (25). Considering the adverse impacts of osteoporosis and osteoarthritis on HRQOL, particularly among women, it is essential to expand strategies aimed at increasing physical activity levels (29, 30). Such interventions could include aquatic exercise, land-based exercise, t’ai chi, and yoga, among others (31). These activities may be particularly beneficial for women at risk of developing osteoporosis and osteoarthritis due to aging and wear after menopause. Promoting physical activity may facilitate reductions in the incidence and severity of these conditions, leading to improved overall health outcomes.

In this study, injuries are associated with decreased HRQOL, particularly in terms of self-care, daily activities, and pain/discomfort domains of the EQ-5D instrument. A literature review investigating changes in quality of life after traffic accidents found that quality of life decreases regardless of injury severity and that recovery takes a long time (7). De Munter et al. (32) confirmed improvements in mobility, pain/discomfort, and usual activity as elements of post-injury recovery, and they identified age, gender, pre-injury frailty, and pre-injury EQ-5D as potential prognostic factors. In particular, like the results of this study, age and gender are significant predictors, emphasizing the need for various educational programs to prevent injuries in middle-aged or older adults women. Moreover, pain resulting from injuries can often lead to chronic musculoskeletal pain. When pain is left unmanaged, functional limitations may arise, leading to a deterioration in HRQOL and a negative perception of one’s subjective health status (32, 33). Therefore, active pain management is crucial in preventing and managing chronic pain and its associated negative consequences. Ultimately, to evaluate injured patients, it is important to accurately identify pain, the range of activity limitations, and the degree of self-care; educational endeavors and interventions focused on these elements should be applied in medical institutions and communities.

This study found no statistically significant associations between anxiety/depression, mobility, and HRQOL. This finding contradicts with previous research which highlighted the role of anxiety and depression in influencing physical activity levels and diminishing overall well-being (34, 35). The present study’s participants were young and middle-aged women, and the results revealed that mental health issues, such as stress, anxiety, and depression, were more prevalent in the middle-aged group. These issues were also found to be related to mobility, as discomfort or pain can lead to a loss of physical self-esteem, which in turn may lead to depression (9, 35, 36). Thus, it is crucial to adopt a nuanced approach to addressing mental health issues—such as stress, anxiety, and depression—among injured women. By tailoring interventions and support to address specific risk factors, healthcare professionals can help injured women manage these issues more effectively, ultimately improving their HRQOL and promoting better outcomes for their recovery. The study findings accurately identify the importance of targeted interventions and support, and they highlight the need to consider factors, such as age and degree of mobility, when designing interventions for injured women. Additionally, for middle-aged women, it is important to improve HRQOL and subjective health status through daily exercise to enhance physical ability and prevent injuries. This can help medical institutions and communities manage injuries more effectively and promote faster recovery. By adopting active approaches to injury management and encouraging regular exercise, healthcare professionals can help middle-aged women maintain their physical and mental health, ultimately improving their overall quality of life.

This study had some limitations. First, this study relied on secondary data analysis, which resulted in limitations regarding the availability of detailed information on various characteristics that influence women’s injury experiences. Second, despite being a representative sample as a cross-sectional study, it is difficult to explain changes in temporal context. Therefore, future studies are necessary to comprehensively evaluate the specific characteristics associated with injuries in adult women. Third, the severity of the injury experienced, the area of the injury, the duration of treatment, whether surgery was performed, underlying diseases that were not investigated, and accompanying complications were not taken into consideration. In future studies, it is necessary to consider additional variables that may have an impact on injuries. In addition, the EQ-5D is a commonly used tool to measure HRQOL. However, few studies have investigated nationwide data to determine associations between injury and HRQOL among women. To address this gap, the researchers analyzed a nationally representative cohort to explore the relationship between HRQOL and injury experience among Korean women.

## 5. Conclusion

This study analyzed nationally representative data from the KNHANES to investigate the relationship between HRQOL and injury among Korean women. The findings suggest that injury is significantly associated with women’s HRQOL, and degenerative musculoskeletal disorders increase the risk of injury. It is important to consider factors such as age and degree of mobility when designing interventions for injured women. Education and preventive interventions are also needed to increase safety awareness for injury prevention for middle-aged women. Daily exercise can improve HRQOL and subjective health status, enhance physical ability, and prevent injuries. Additionally, women who have experienced injuries need accurate assessment of pain and discomfort and customized education to promote self-care for recovery. This can help medical institutions and communities manage injuries more effectively and promote faster recovery.

## Data availability statement

Publicly available datasets were analyzed in this study. This data can be found here: The data that support the findings of this study are available from the corresponding author upon reasonable request. The datasets used and/or analyzed during the current study are publicly available upon request via https://knhanes.kdca.go.kr/knhanes/sub03/sub03_02_05.do.

## Author contributions

All authors listed have made a substantial, direct, and intellectual contribution to the work and approved it for publication.
